# Establishment and validation of a novel disulfidptosis-related immune checkpoint gene signature in clear cell renal cell carcinoma

**DOI:** 10.1007/s12672-024-01105-x

**Published:** 2024-06-21

**Authors:** Lihuan Du, Nan Zhang, Bohan Wang, Wei Cheng, Jiaming Wen

**Affiliations:** 1 Department of Urology, The Second Affiliated Hospital of Zhejiang University, NO. 88 Jiefang Road, Hangzhou, 310009 China; 2 Department of Urology, Traditional Chinese Medicine Hospital of Longyou, Longyou, 324400 Quzhou China

**Keywords:** Disulfidptosis, Immune checkpoint, Clear cell renal carcinoma, Gene signature, Prognostic

## Abstract

**Background:**

Clear cell renal cell carcinoma (ccRCC) is the most prevalent subtype of renal tumors and is associated with a unfavorable prognosis. Disulfidptosis is a recently identified form of cell death mediated by disulfide bonds. Numerous studies have highlighted the significance of immune checkpoint genes (ICGs) in ccRCC. Nevertheless, the involvement of disulfidptosis-related immune checkpoint genes (DRICGs) in ccRCC remains poorly understood.

**Methods:**

The mRNA expression profiles and clinicopathological data of ccRCC patients were obtained from The Cancer Genome Atlas and Gene Expression Omnibus (GEO) databases. The associations between disulfidptosis-related genes (DRGs) and immune checkpoint genes (ICGs) were assessed to identify DRICGs. Cox regression analysis and least absolute shrinkage and selection operator (LASSO) analysis were conducted to construct a risk signature.

**Results:**

A total of 39 differentially expressed immune-related candidate genes were identified. A prognostic signature was constructed utilizing nine DRICGs (CD276, CD80, CD86, HLA-E, LAG3, PDCD1LG2, PVR, TIGIT, and TNFRSF4) and validated using GEO data. The risk model functioned as an independent prognostic indicator for ccRCC, while the associated nomogram provided a reliable scoring system for ccRCC. Gene set enrichment analysis indicated enrichment of phospholipase D, antigen processing and presentation, and ascorbate and aldarate metabolism-related signaling pathways in the high-risk group. Furthermore, the DRICGs exhibited correlations with the infiltration of various immune cells. It is noteworthy that patients with ccRCC categorized into distinct risk groups based on this model displayed varying sensitivities to potential therapeutic agents.

**Conclusions:**

The novel DRICG-based risk signature is a reliable indicator for the prognosis of ccRCC patients. Moreover, it also aids in drug selection and correlates with the tumour immune microenvironment in ccRCC.

**Supplementary Information:**

The online version contains supplementary material available at 10.1007/s12672-024-01105-x.

## Introduction

Renal cell carcinoma (RCC) is the most fatal malignant urinary system tumour worldwide [1], and ccRCC is the predominant histology of RCC with an increasing prevalence and is the leading cause of cancer-related death [2]. The main therapeutic option for patients with ccRCC is surgery, with 30% of ccRCC patients experiencing recurrence, cancer progression and even metastasis following radical nephrectomy [3]. ccRCC is relatively resistant to conventional radiotherapy and chemotherapy [4]. The overall prognosis of patients with ccRCC remains poor, especially for metastatic renal cell carcinoma (mRCC).

RCC tissues exhibit infiltration by various inflammatory cell types, including natural killer cells, dendritic cells, T cells, and macrophages, indicating the potential efficacy of immunotherapy as a treatment option for clear cell renal cell carcinoma (ccRCC). Tumors, including ccRCC, have the ability to engage immune checkpoint (IC) pathways as a mechanism to evade detection by the immune system [5]. Therefore, immune checkpoint inhibitors (ICIs), which involve antibodies and target different proteins of the immune checkpoint pathway, such as CTLA-4, CD86/B7-2, CD80/B7-1, PD-1, PD-L1/B7-H1 and PD-L2/B7-DC, can activate the tumour immune system and are now considered a potential treatment strategy for mRCC [6]. However, approximately 40–60% of patients still have intrinsic resistance to ICIs and experience no benefit from ICI treatments [7, 8]. Thus, identifying novel ICG-related biomarkers is of great importance in developing candidate immunotherapeutic targets for ccRCC.

Disulfidoptosis represents a newly identified form of cellular demise triggered by an overabundance of intracellular disulfides, setting it apart from other forms of cell death associated with oxidative stress, including ferroptosis, apoptosis, and necroptosis [9].Cystine transporter solute carrier family 7 member 11 (SLC7A11), cystine uptake and cystine reduction to cysteine, glucose starvation, and NADPH pool depletion are considered key regulators of disulfidptosis. However, the interaction between DRGs and ICGs in ccRCC remains unknown. Moreover, it is imperative to elucidate the involvement of disulfidptosis-related immune checkpoint genes in ccRCC. The present study aims to establish a risk signature based on immune checkpoint genes, assess its correlation with disulfidptosis, and explore its prognostic implications and underlying mechanisms in ccRCC patients.

## Materials and methods

### Data acquisition and DRICG identification

 The mRNA expression levels of 537 ccRCC patients and the relevant clinicopathologic data were downloaded from The Cancer Genome Atlas (TCGA) database up to 21 April 2022, including 539 ccRCC samples and 72 paracancerous samples. RNA sequencing data were transformed from fragments per kilobase million (FPKM) format to transcripts per million (TPM) reads. The RNA-seq data and related clinical information of an ccRCC cohort were also retrieved from the GEO database (GSE29609). A total of 79 ICGs and 23 DRGs were retrieved from previous studies [9, 10], which are presented in Supplementary Table 1 and Table 2. Then, correlation analysis between DRGs and ICGs was conducted to identify the particular ICGs (pICGs) that were highly correlated with DRGs (cor > 0.3, p < 0.05), hereafter referred to as DRICGs.

### Construction and validation of the DRICG-related prognostic risk model

Univariate Cox regression and least absolute shrinkage and selection operator (LASSO) regression analysis were employed to develop the prognostic gene signature. Survival-related genes were identified with a cut-off P value of 0.05. The risk score of each patient was calculated using the following formula: risk score = ∑^6^_i_ Xi × Yi (X: regression coefficients, Y: normalized gene expression level). The ccRCC patients were stratified into a high-risk group and low-risk group in accordance with the median risk score. Kaplan–Meier curves were employed to evaluate the different prognoses of the two risk groups. The “survival”, “survminer” and “timeROC” R packages were used to conduct 1-year, 3-year and 5-year receiver operating characteristic (ROC) curve analyses and assess the predictive power of the risk model.

A ccRCC cohort from the GEO dataset (GSE29609) was employed for the external validation of the prognostic performance of this risk signature in ccRCC. The risk score was calculated by the same formula that was used for the TCGA cohort. The patients in the GSE29609 cohort were also divided into low- and high-risk subgroups according to the median risk score from the TCGA cohort.

### Nomogram establishment and clinical relevance investigation

Univariate and multivariate Cox regression analyses were conducted to investigate whether the risk scores and other clinical variables were independent prognostic factors in ccRCC patients. Moreover, a nomogram based on the Cox regression results was established to evaluate the probability of 1-,3-, and 5-year overall survival (OS) for ccRCC patients. The C-index and calibration curve were utilized to assess the predictive performance of the nomogram. Then, a chi-square test was performed to explore the correlation between the DRICG-based signature and clinicopathologic data, including age, gender, tumor grade and stage. In addition, survival stratification analysis was employed to determine the prognostic value of the risk signature in different ccRCC subgroups.

### Gene set enrichment and immune cell infiltration analyses

Gene set enrichment analysis (GSEA) analysis was conducted to investigate the potential molecular mechanisms and downstream signalling pathways among the low- and high-risk groups. Statistical significance was set at a P value < 0.05 and FDR < 25%. In addition, the relationship between the infiltration level of 6 specific immune cell subsets (B cells, CD4 + T cells, CD8 + T cells, macrophages, neutrophils and dendritic cells) and the above 9 DRICGs was evaluated based on the TIMER database to further explore the role of DRICGs in ccRCC.

### Drug sensitivity analysis

The Genomics of Drug Sensitivity in Cancer (GDSC) database was utilized to predict the difference in drug sensitivity between the two risk groups. The half-maximal inhibitory concentration (IC50) of different drugs was used to analyse the drug sensitivity of ccRCC via the pRRophetic package. The sensitivity indicators were expressed as IC50 values.

### Statistical analysis

**Fig. 1:**
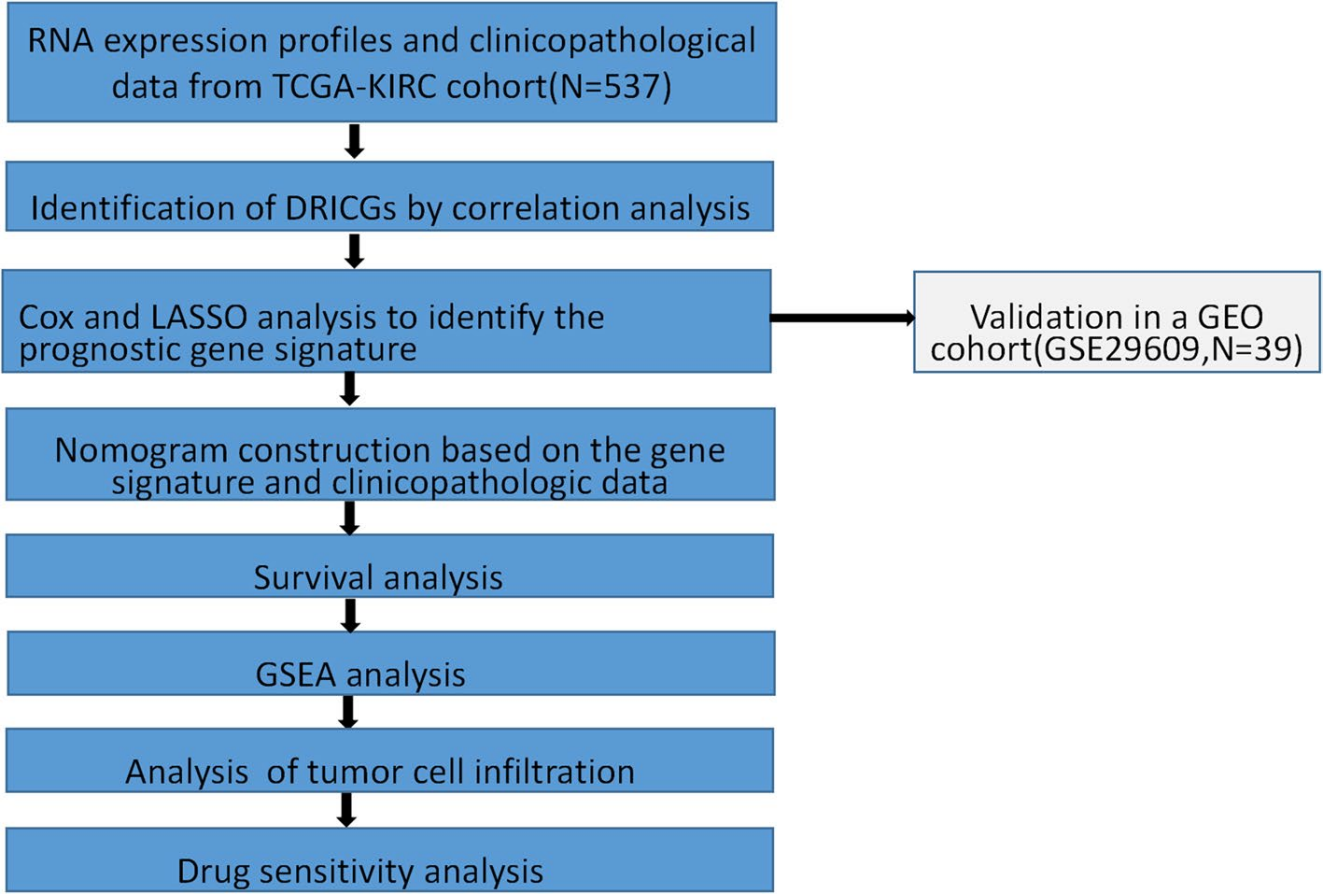
The workflow diagram of this study

All statistical analyses were performed with R software (v4.1.2). The log-rank test was employed for the Kaplan–Meier survival analysis. Student's t test and the Pearson chi-square test were employed in the two-group comparisons. A P value less than 0.05 was considered statistically significant if not specified. The flow diagram of this study is shown in Fig. 1.

## Results

### Identification of DRICGs

In total, we determined that 39 ICGs (ADORA2A, BTN2A1, BTNL9, CD209, CD226, CD27, CD276, CD28, CD40, CD47, CD70, CD80, CD86, CD96, HLA-A, HLA-B, HLA-C, HLA-DMA, HLA-DPA1, HLA-DPB1, HLA-DQA1, HLA-DRA, HLA-DRB1, HLA-E, HLA-F, LAG3, LGALS9, PDCD1, PDCD1LG2, PVR, SIRPA, TIGIT, TNFRSF14,TNFRSF18,TNFRSF4,TNFRSF9,TNFSF14,TNFSF9 and VTCN1) were closely correlated with disulfidptosis-related genes using a correlation analysis (Supplementary Table 3).Therefore, these hub genes were defined as the DRICGs for further studies.

### Establishment of the DRICG-based signature

**Fig. 2:**
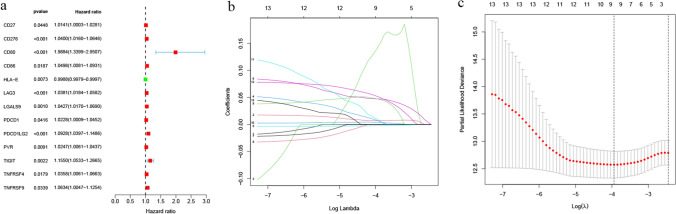
Identification of the candidate prognostic DRICGs. **a** Univariate Cox regression analysis of OS for 13 DRICGs with P < 0.05. **b** LASSO regression of the 9 OS-related DRICGs. **c** Partial likelihood deviance is plotted versus log (λ)

A total of 13 DRICGs (CD27, CD276, HLA-E, CD80, CD86, LAG3, LGALS9, PVR, PDCD1, PDCD1LG2, TIGIT, TNFRSF9 and TNFRSF4) were identified as prognostic genes according to the criterion of P < 0.05 (Fig. 2a) in an univariate Cox regression analysis. The candidate genes were subsequently inputted into the LASSO algorithm, resulting in the construction of a 9-gene signature based on the optimal λ value for prognostic prediction in ccRCC patients. (Fig. 2b, c). This formula was used to calculate the risk score for every patient: risk score = (0.007*CD276 expression level) + (0.142*CD80 expression level) + (0.007*CD86 expression level) + (− 0.002* HLA-E expression level) + (0.047*LAG3 expression level) + (0.047* PDCD1LG2 expression level) + (0.004*PVR expression level) + (0.009*TIGIT expression level) + (0.051* TNFRSF4 expression level). A median risk score was used to divide patients with ccRCC into high-risk and low-risk groups.

**Fig. 3:**
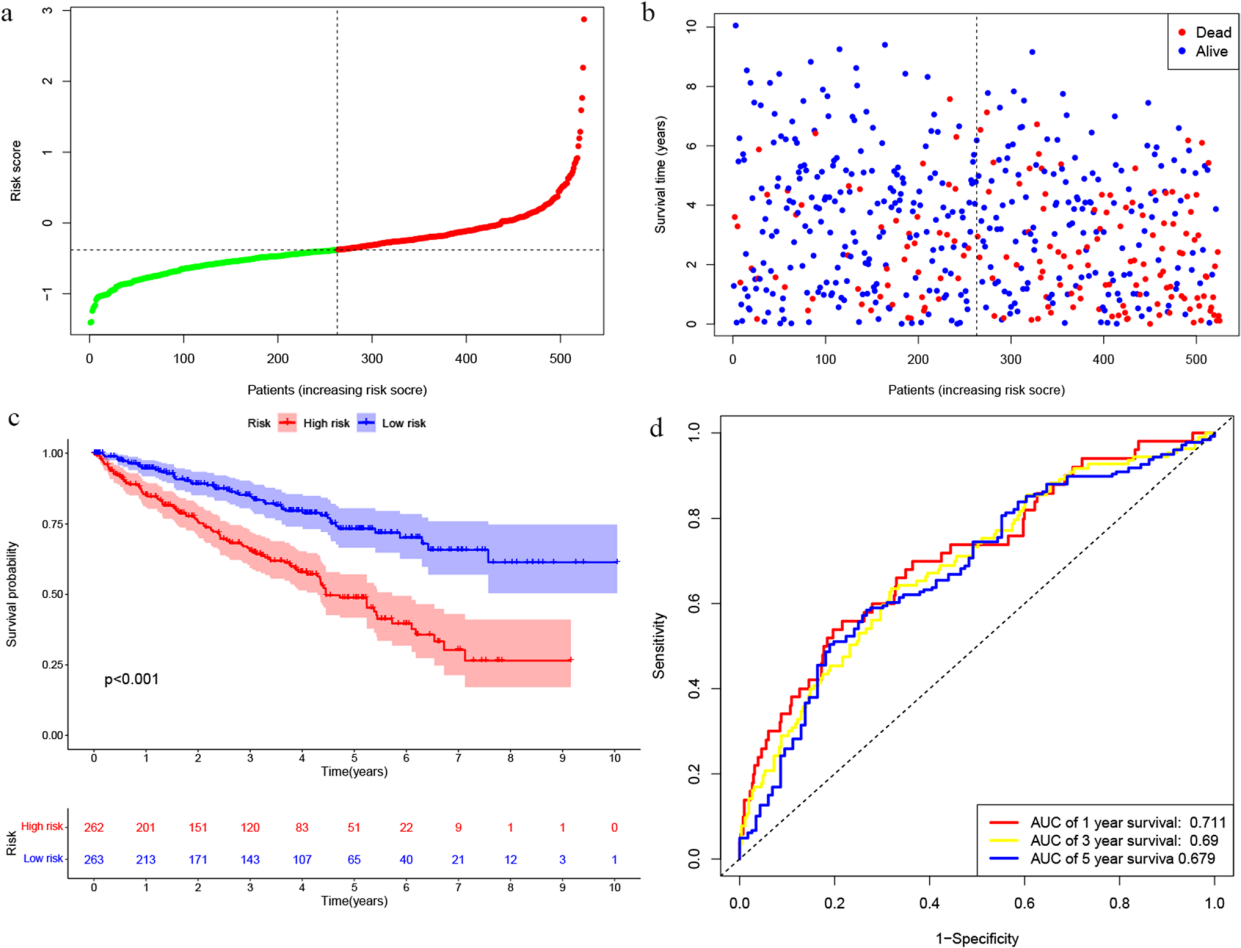
Construction and evaluation of the DRICG-based risk signature in ccRCC. **a** Distribution of each patient based on different risk scores. **b** The survival status distribution for patients based on different risk scores. **c** The Kaplan–Meier curve demonstrates that individuals classified in the high-risk group exhibited markedly inferior OS outcomes compared to those categorized in the low-risk group (P < 0.001). **d** Time-dependent ROC curves demonstrate the predictive accuracy of the risk score for the 1-year, 3-year and 5-year survival of ccRCC patients

A significant difference between low- and high-risk ccRCC patients was found in the survival times (P < 0.001). These findings suggest a negative relationship between the risk score and prognosis for ccRCC patients (Fig. 3a–c). The area under the curve (AUC) values of 0.711 for 1-year survival, 0.69 for 3-year survival and 0.679 for 5-year survival were observed using time-dependent ROC analysis of the prognostic risk model that incorporates the DRICG-based signature (Fig. 3d).

### External validation of the risk signature

**Fig. 4:**
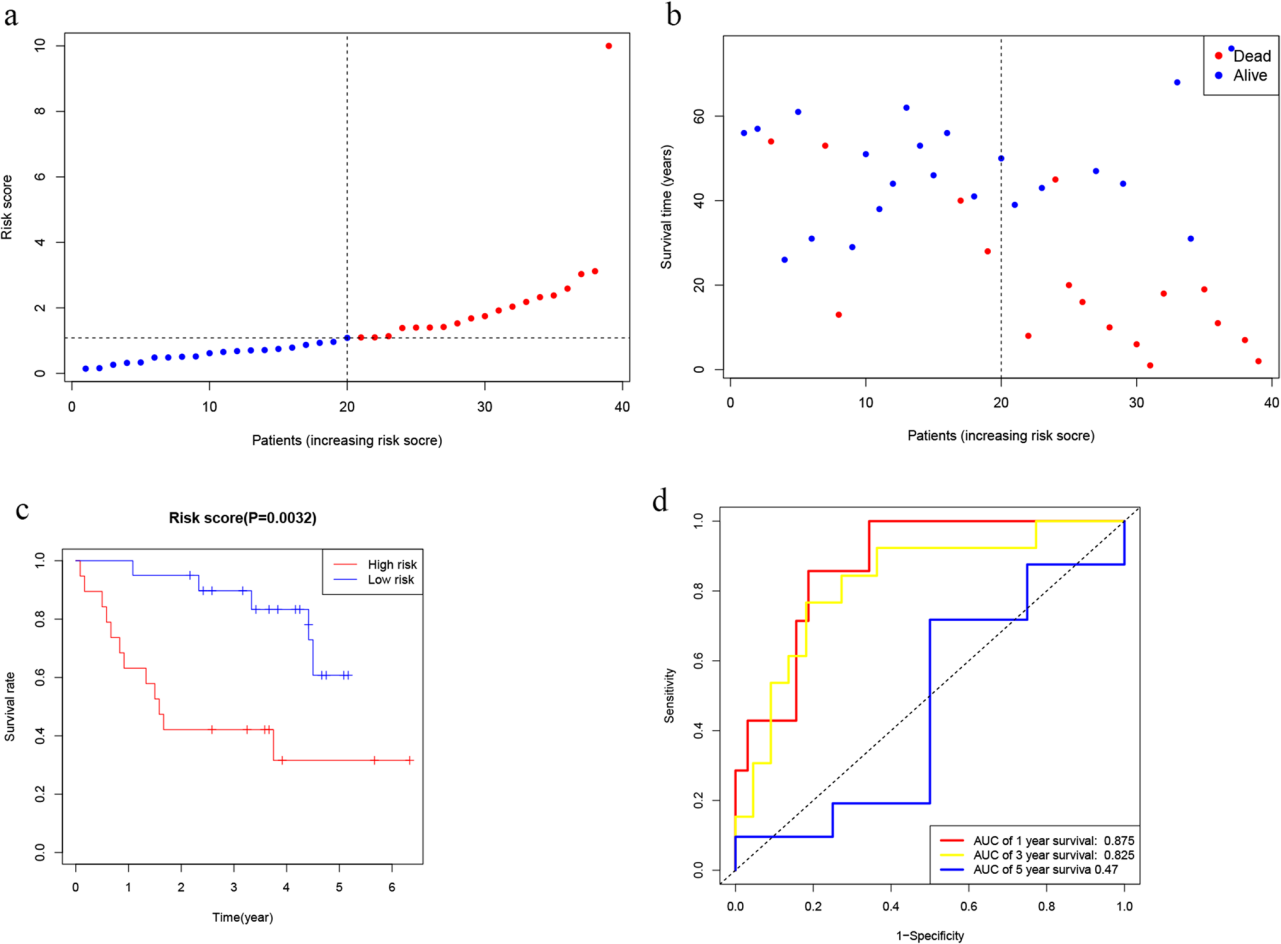
Validation of the risk model in the GEO cohort. **a** Distribution of patients and median risk score. **b** PCA plot. **c** Comparison of the OS between low- and high-risk groups in ccRCC. **d** AUC of time-dependent ROC curves in the GEO cohort

The validation set consisted of 39 patients with ccRCC from the GEO dataset GSE29609. In the GEO cohort, 19 patients were classified as high-risk, while the remaining 20 were classified as low-risk based on the median risk score in the TCGA cohort. The results of our study indicate that low-risk subgroups had significantly lower mortality rates than high-risk subgroups (Fig. 4a, b). Kaplan–Meier analysis also revealed a statistically significant difference in survival rates between low-risk and high-risk groups (P = 0.0032, Fig. 4c). Furthermore, the prognostic signature had an AUC of 0.875 at 1 year, 0.825 at 2 years, and 0.47 at 5 years. (Fig. 4d).

### Independent prognostic ability of the risk model

**Fig. 5:**
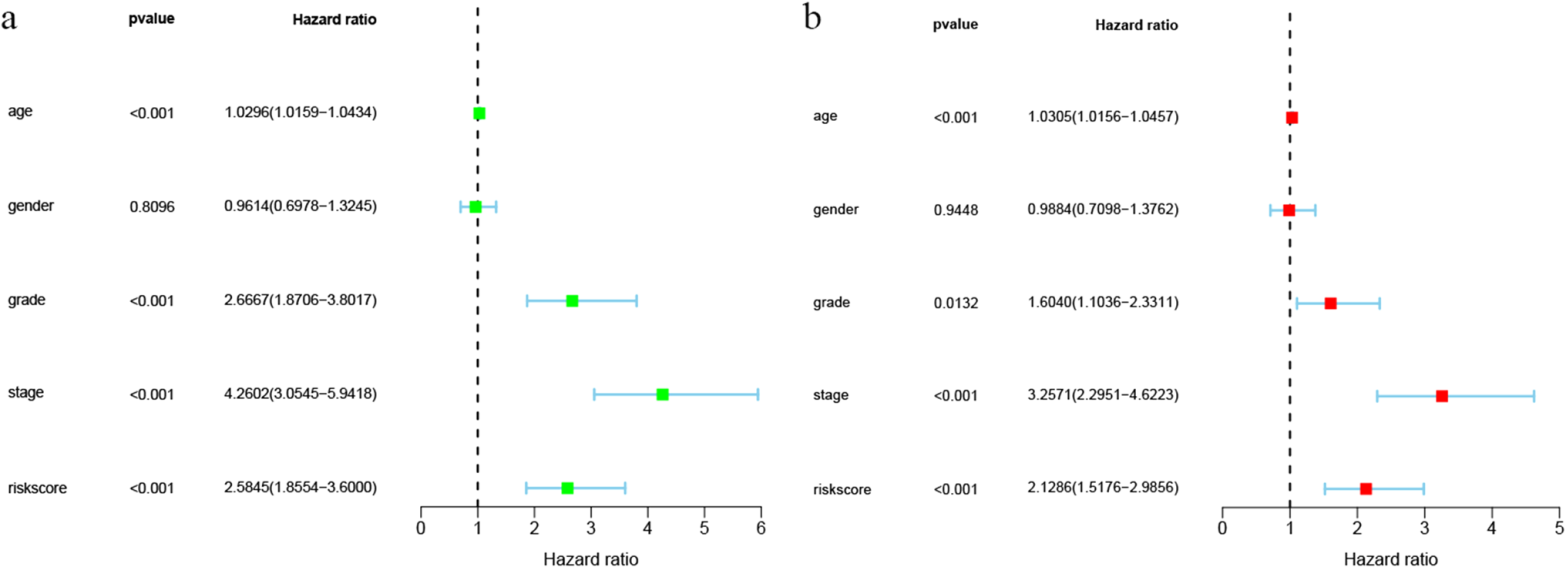
Univariate and multivariate Cox regression analyses of clinicopathologic factors and the gene signatures in ccRCC patients. **a** Univariate Cox regression analysis for the correlations between the risk score and clinicopathological factors, including age, gender, tumour grade and stage; **b** The correlations between the risk score and clinicopathological factors in survival prediction by multivariate Cox regression analysis

As shown in Fig. 5a, we observed that the risk score, age, tumour grade and stage were all positively associated with the survival of ccRCC patients using univariable Cox analysis (P < 0.05), but gender was not. Multivariate analysis further confirmed that the risk score, age, tumour grade and stage were independent risk factors related to prognosis (P < 0.05) (Fig. 5b).

### Nomogram construction based on the risk signature

**Fig. 6:**
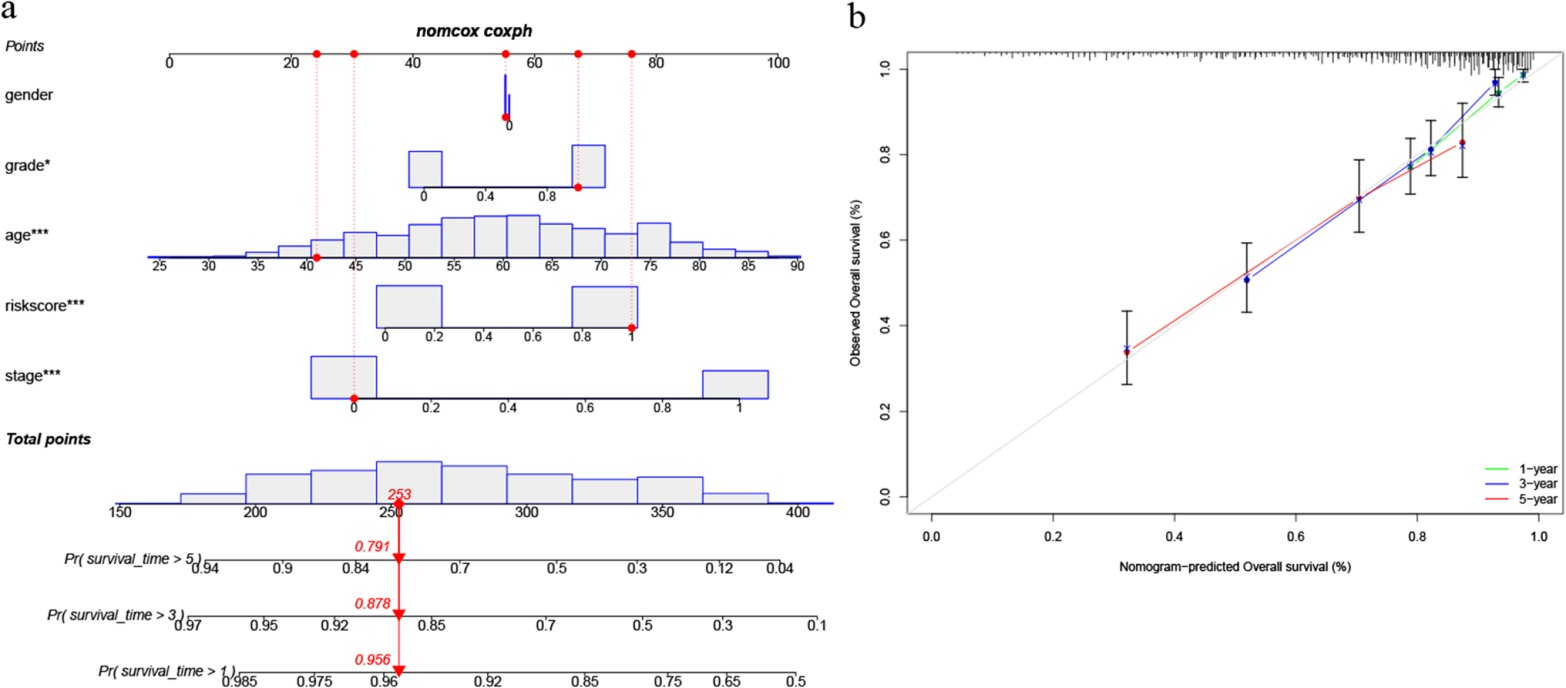
Construction and evaluation of a nomogram based on the risk score and other clinicopathologic parameters. **a** The nomogram predicting the 1-, 3- and 5-year OS of each patient; **b** The calibration plots for the probability of 1-, 3- and 5-year OS of ccRCC patients

As shown in Fig. 6a, the established nomogram graphically predicted the 1-, 3- and 5-year survival probability of ccRCC patients. The calibration curve indicated that the nomogram was well calibrated, with the actual survival of each patient close to the predicted probabilities (Fig. 6b). The concordance index (C-index) of 0.76 indicates a high level of agreement between the observed and nomogram-derived estimates of survival probability (1-, 3-, and 5-year OS). These results indicated that the constructed nomogram was a reliable scoring system.

### Correlation analysis between the gene signature and clinicopathologic characteristics of ccRCC

**Fig. 7:**
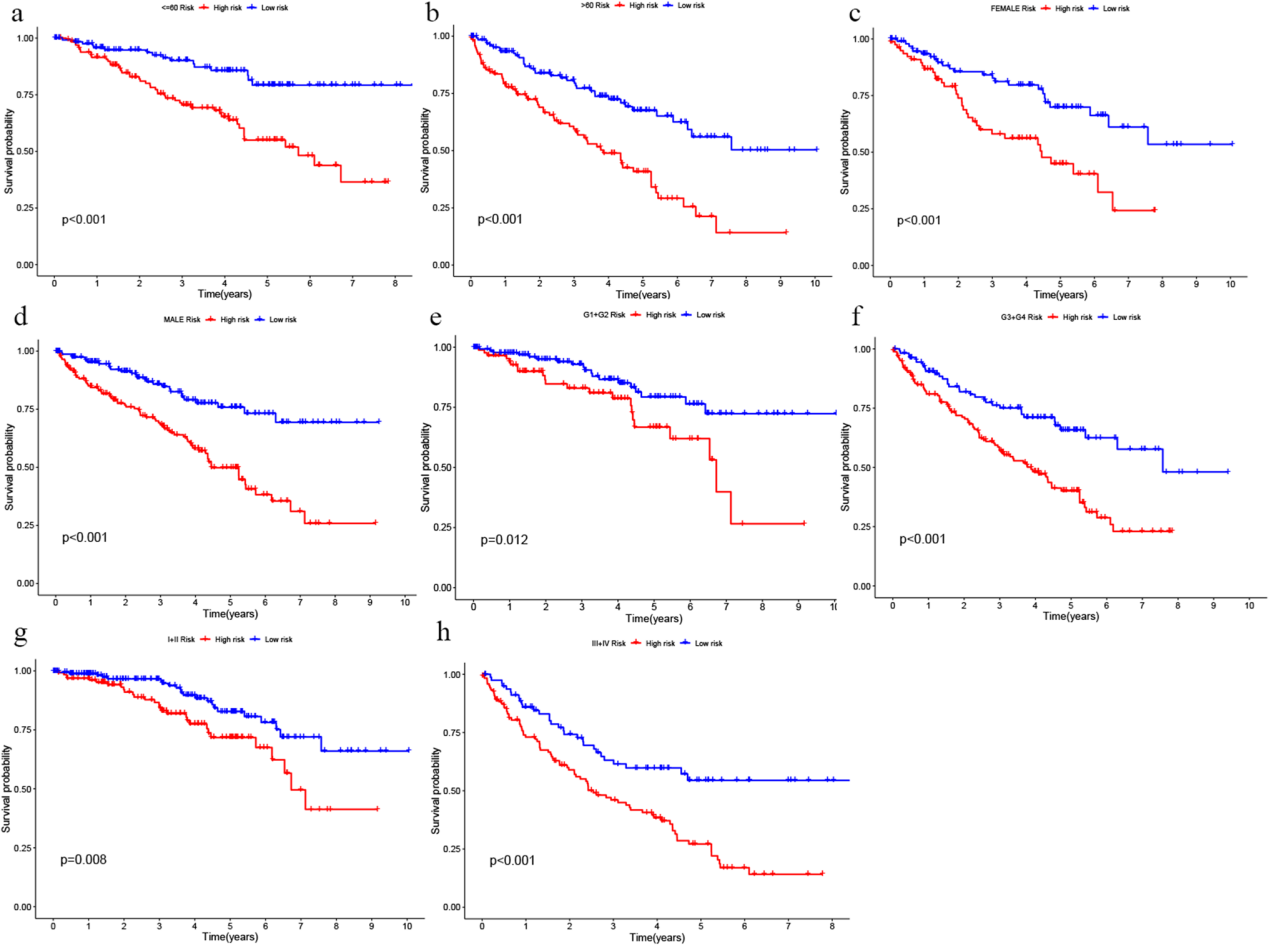
Kaplan–Meier survival analysis for the prediction of the risk model of the survival of ccRCC patients, stratified by age, gender, grade and stage. **a**, **b** Patients aged < 60 years and ≥ 60 years subgroups; **c**, **d** Male and female subgroups; **e**, **f** Tumour grade 1–2 and grade 3–4 subgroups; **g**, **h** Tumour stage I-II and stage III-IV subgroups

Survival stratification analysis indicated that the DRICG-based signature had noteworthy performance in survival prediction in subgroups of age > 60(P < 0.001), age <  = 60 (P < 0.001), female (P < 0.001), male (P < 0.001), grade G1-G2 (P = 0.012), grade G3-G4 (P < 0.001), stage I-II (P = 0.008) and stage III-IV (P < 0.001) (Fig. 7).

### GSEA analysis

**Fig. 8:**
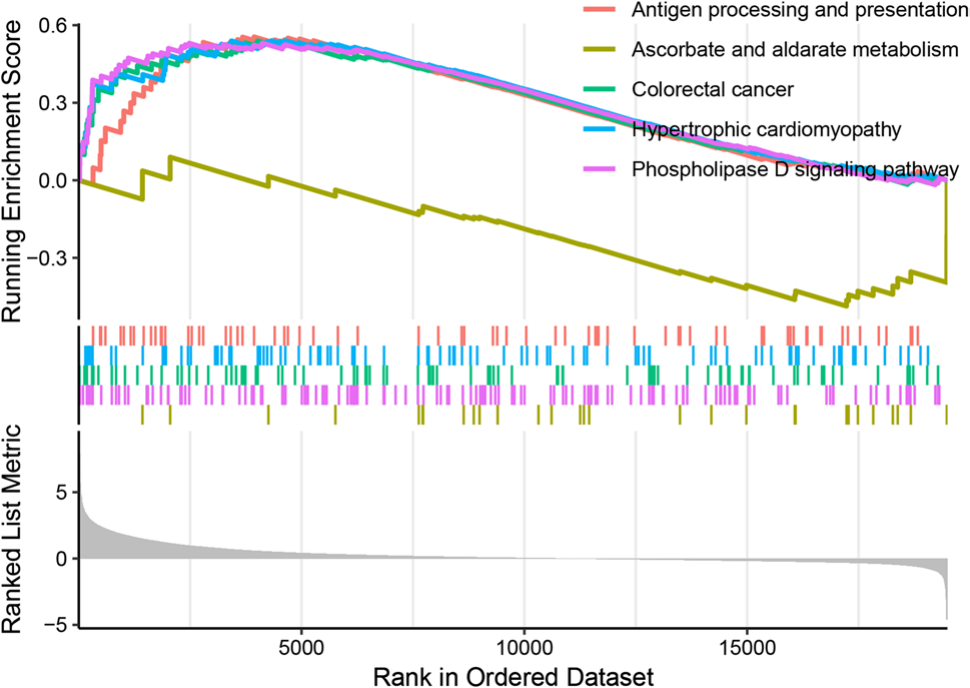
GSEA revealed that the high-risk group exhibited enrichment of multiple signaling pathways

The GSEA results demonstrated that three metabolism- and tumour-related pathways, including the phospholipase D signalling pathway, antigen processing and presentation, and ascorbate and aldarate metabolism-related signalling pathways, were enriched in the high-risk group (Fig. 8).

### Immune infiltration analysis

**Fig. 9:**
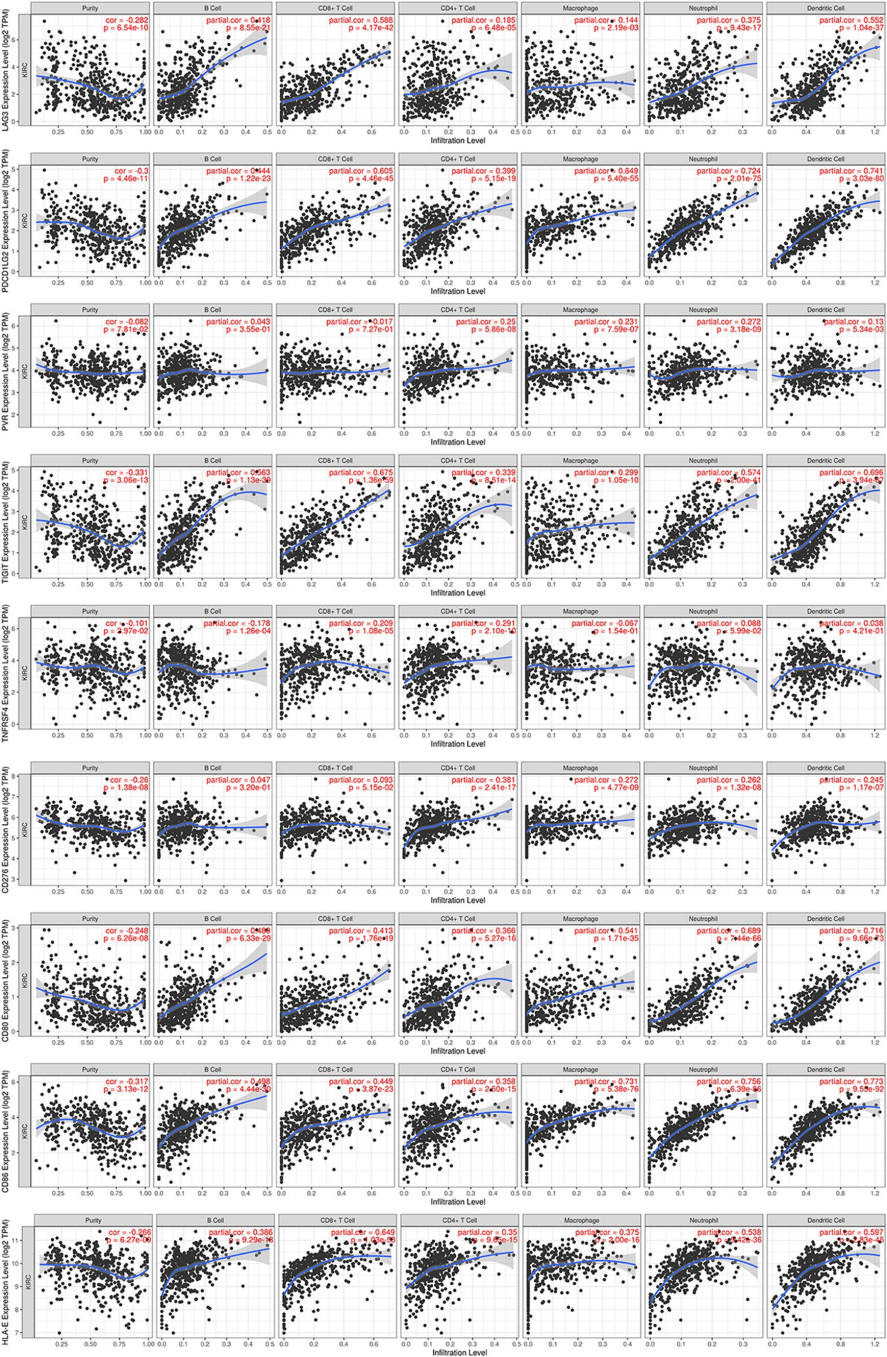
Correlations between tumour immune cell infiltration and the nine prognostic DRICGs

The study revealed significant positive associations between CD80, CD86, PDCD1LG2, and HLA-E with B cells, CD8 + T cells, CD4 + T cells, neutrophils, macrophages, and dendritic cells. Furthermore, TIGIT showed positive correlations with B cells, neutrophils, CD4 + T cells, CD8 + T cells, and dendritic cells, while LAG3 was also positively linked to multiple immune cell types including B cells, neutrophils, CD8 + T cells, and dendritic cells. CD276 was found to be positively associated with CD4 + T cells. Notably, TNFRSF4 and PVR did not demonstrate statistically significant relationships with the six predominant immune cell types. (Fig. 9).

### Drug sensitivity analysis

**Fig. 10:**
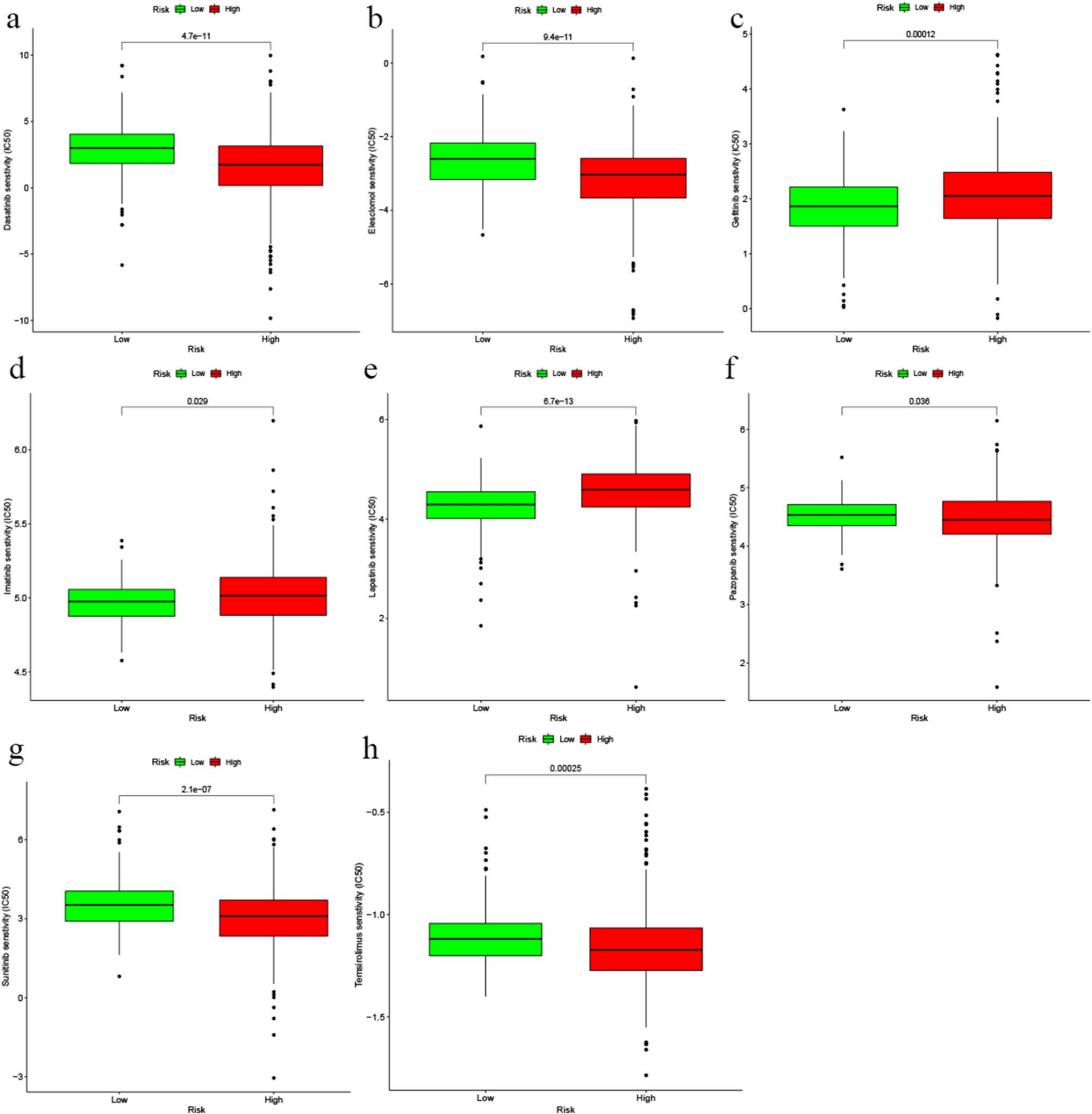
The correlations between the DRICG-based risk score and the sensitivity of 8 candidate drugs for ccRCC patients. **a** dasatinib; **b** elesclomol; **c** gefitinib; **d** imatinib; **e** lapatinib; **f** pazopanib; **g** sunitinib; **h** elesclomol

We observed that ccRCC patients in the high-risk group were more sensitive to gefitinib, imatinib and lapatinib than those in the low-risk group. Conversely, ccRCC patients in the low-risk group might achieve better outcomes after receiving dasatinib, sunitinib, elesclomol, pazopanib and temsirolimus than those in the high-risk group (Fig. 10).

## Discussion

Renal cell carcinoma is considered a type of immunogenic tumour because of its high immune infiltration and low mutational burdens and because it is prototypically responsive to immunotherapies such as ICIs [11]. ICIs have revolutionized the treatment of RCC and have become the first-line treatment choice for mRCC [12]. However, the population that experiences an immune-benefit is still limited, largely due to a lack of specific biomarkers predicting benefit-based patients [13]. Therefore, there is an urgent need to explore new therapeutic ICG targets that are also new markers to predict the immune efficacy and prognosis of ccRCC patients.

Disulfidptosis, recently demonstrated by Liu X et al., is a new form of disulfide-dependent cell death triggered by the aberrant accumulation of intracellular disulfides, which results in disulfide stress that is highly toxic to cells [9]. It is an unexpected mechanism of cell death and might offer novel directions and targets to treat malignancy. To date, few studies have comprehensively investigated the connections between ICGs and DRGs, the role of DRICGs and their potential mechanisms in ccRCC.

In the present study, we first extracted 79 ICGs and 23 DRGs from previous reports [9, 10]. Thirty-nine ICGs were identified as hub genes that correlated with disulfidptosis-related genes and were termed DRICGs. Then, a signature of nine DRICGs (CD276, HLA-E, CD80, CD86, LAG3, PVR, PDCD1LG2, TIGIT and TNFRSF4) was constructed that was associated with the survival rates of ccRCC. Specifically, ccRCC patients in the high-risk group generally exhibited worse overall survival than those in the low-risk group, which was externally validated by the GEO dataset, and the time-dependent ROC curve further indicated the good predictive power of this risk model. The risk score was higher in male patients with a more advanced stage and tumour grade. The current risk model in clinical practice, the International Metastatic Renal Cell Carcinoma Database Consortium (IMDC) model, was based on clinical factors and is commonly used for prognostic stratification of mRCC patients. The interpatient variability within these subgroups divided by the IMDC model is high [14].

Notably, the nomogram composed of the prognostic signature as well as age, grade and stage further indicated that older age, higher tumour stage and grade, and higher risk score were independent risk factors for poorer prognosis. The C-index and calibration curve analysis confirmed its good discrimination. Compared to traditional prognostic models such as the IMDC model, the established nomogram was a joint model of molecular subtypes and clinical factors, which may improve the accuracy of prognostic prediction. The stratified survival analysis showed that patients in the high-risk group suffered a significantly poorer OS than those in the low-risk group for the representative cohorts, including different age, gender, grade and stage subgroups. Collectively, these findings validate the prognostic predictive capability of the DRICG-based risk score in ccRCC.

CD276, also known as B7-H3, is a transmembrane protein and immune checkpoint molecule within the B7 family that serves as the target for CAR-T cells [15, 16]. It is overexpressed in a variety of tumours such as prostate cancer and ovarian cancer, and its blockade can inhibit tumour metastasis by enhancing CD8 + T-cell-mediated antitumour immunity [17, 18]. HLA-E belongs to the nonclassical class of HLA-I antigens and negatively regulates the cytotoxic function of CD8 + T cells and NK cells. It was also found to be related to tumour immune escape in RCC [19, 20]. PDCD1LG2 (programmed cell death 1 ligand 2, also called PDCD1 ligand 2, PD-L2, or CD273) is an independent prognostic factor and is associated with the intratumoral infiltration of CD8 + TILs in colon cancer [21]. A member of the nectin-like protein family, PVR (poliovirus receptor, CD155) is involved in several cellular processes, such as adhesion, migration, and proliferation. It can enhance the antitumour response, and the TIGIT-PVR immune checkpoint axis is considered a potential immunotherapy target [22]. The immune receptor TIGIT, which contains T-cell immunoreceptor with immunoglobulin and tyrosine-based inhibitory motif domains, is implicated in the inhibition of T-cell activity through its interaction with CD155, thereby contributing to the promotion of tumorigenesis [23]. Additionally, the immune checkpoint proteins CD80 (B7-1) and CD86 (B7-2) are expressed on the surfaces of both tumor and immune cells, serving as significant prognostic biomarkers in various cancers, including bladder cancer and non-small cell lung carcinoma [24, 25]. LAG-3, also known as CD223, is an immune checkpoint receptor protein that plays a crucial role in maintaining microenvironment stability through immune regulation. It is implicated in immune escape and has a strong correlation with tumour initiation and advancement [26]. TNFRSF4, also known as OX40 or CD134, is involved in the differentiation, proliferation, and survival of T cells by activating the PI3K/PKB, NFAT, and NF-kappa-B pathways. TNFRSF4 is considered an immunotherapy target of tumours [27–29].

Renal cell carcinoma is fundamentally a metabolic disorder distinguished by a reconfiguration of energetic metabolism, including alterations in the distribution of metabolic flux through glycolysis, as well as dysfunction in mitochondrial bioenergetics and OxPhox, along with perturbations in lipid metabolism [30–36]. Recent studies have highlighted the significance of disulfidptosisis as a key modulator of cellular metabolism [37]. In this study, the GSEA results also demonstrated that the DRICG-based signature was mainly participated in metabolism- and cancer-related pathways, including the phospholipase D signalling pathway, antigen processing and presentation, and ascorbate and aldarate metabolism-related signalling pathways. Emerging evidence indicated that the activation of metabolic pathways is crucial in the regulation of angiogenesis and inflammatory responses [38, 39]. These diverse pathways may be the underlying mechanism for the varying prognosis of ccRCC patients in the high- and low-risk groups.

Renal cell carcinoma is one of the most immune-infiltrated tumors [40–42]. In our study, TIMER database analysis indicated that seven of the identified DRICGs, excluding TNFRSF4 and PVR, had a close relationship with immune cell infiltration. Importantly, four DRICGs (CD80, CD86, PDCD1LG2 and HLA-E) showed a positive correlation with the six common immune cells (B cells, CD8 + T cells, CD4 + T cells, macrophages, neutrophils and dendritic cells). The other three DRICGs (TIGIT, LAG3 and CD276) also had a positive association with some of the immune cells. These results uncovered the possible mechanism by which the DRICG-based gene signature regulated tumour progression.

Although great advances have been made in the drug treatments of ccRCC in recent years, there is still an urgent need to distinguish responders and nonresponders to these potential drugs. Multiple studies suggested that characteristics of the tumor microenvironment play a significant role in influencing tumor biology and the efficacy of systemic therapy [43–47]. Moreover, recent studies have shown that disulfidptosis could regulate immune cell infiltration, immunoflogosis and the response to immunotherapy [48]. Therefore, we next screened effective drugs for ccRCC patients in the high- and low-risk groups. The drug sensitivity analysis suggested that ccRCC patients in the high-risk group might benefit from the administration of gefitinib, imatinib and lapatinib, while those in the low-risk group might see better results with dasatinib, sunitinib, elesclomol, pazopanib and temsirolimus. These results suggested that the DRICG-based risk model was an excellent predictor of drug sensitivity and could guide better treatment strategies for ccRCC patients.

Our study has several limitations. First, the prognostic signature construction was mainly based on data from the TCGA database with preliminary verification in the GEO database; thus, more multicentre prospective controlled clinical trials are warranted to verify its clinical utility in the future. Moreover, due to the small sample size of the GEO dataset used in this study, a larger cohort is required to externally validate this model in RCC. In addition, some drugs for RCC such as immune checkpoint inhibitors, were not included in the drug sensitivity analysis because of the R package. More importantly, the mechanisms by which DRICGs regulate the clinical prognosis, immune infiltration and drug sensitivity of ccRCC should be validated by additional experiments with our clinical samples and cell lines.

## Conclusions

In conclusion, we identified for the first time a reliable gene signature based on nine DRICGs that was an independent risk factor for survival prediction in ccRCC. These DRICGs participate in different cancer-related pathways, and might be involved in tumour immunity in ccRCC. Notably, this promising gene signature was also an effective biomarker for the drug selection for ccRCC patients. Therefore, these DRICGs could be potential immunotherapeutic targets for ccRCC. However, further in-depth studies are still needed.

### Supplementary Information

Supplementary file1 (XLSX 9 KB)

Supplementary file2 (XLSX 9 KB)

Supplementary file3 (XLSX 13 KB)

## Data Availability

The authors declare that the data supporting the findings of the current study are provided in the article. Datasets analysed for this work can be obtained from TCGA (https://portal.gdc.cancer.gov/), GEO(http://www.ncbi.nlm.nih.gov/geo/), TIMER (https://cistrome.shinyapps.io/timer/) and GDSC (http://www.cancerrxgene.org/).
